# Supplementary material for: A UMLS-based spell checker for natural language processing in vaccine safety
Source: BMC Med Inform Decis Mak. 2007 Feb 12;7:3. doi: 10.1186/1472-6947-7-3 (PMC1805499; doi:10.1186/1472-6947-7-3)
Supplement: Additional file 13 — RAP application directory. Sets up directory for RAP (RDF application for PHP) source code [file 1472-6947-7-3-S13.gz › rap/api/util/adodb/tests/benchmark.php]

ADODB Benchmarks

php
/\*
V4.52 10 Aug 2004 (c) 2000-2004 John Lim (jlim@natsoft.com.my). All rights reserved.
Released under both BSD license and Lesser GPL library license.
Whenever there is any discrepancy between the two licenses,
the BSD license will take precedence.
Benchmark code to test the speed to the ADODB library with different databases.
This is a simplistic benchmark to be used as the basis for further testing.
It should not be used as proof of the superiority of one database over the other.
\*/
$testmssql = true;
//$testvfp = true;
$testoracle = true;
$testado = true;
$testibase = true;
$testaccess = true;
$testmysql = true;
$testsqlite = true;;
set\_time\_limit(240); // increase timeout
include("../tohtml.inc.php");
include("../adodb.inc.php");
function testdb(&$db,$createtab="create table ADOXYZ (id int, firstname char(24), lastname char(24), created date)")
{
GLOBAL $ADODB\_version,$ADODB\_FETCH\_MODE;
adodb\_backtrace();
$max = 100;
$sql = 'select \* from ADOXYZ';
$ADODB\_FETCH\_MODE = ADODB\_FETCH\_NUM;
//print "<h3ADODB Version: $ADODB\_version Host: *$db->host*   Database: *$db->database*";
// perform query once to cache results so we are only testing throughput
$rs = $db->Execute($sql);
if (!$rs){
print "Error in recordset

";
return;
}
$arr = $rs->GetArray();
//$db->debug = true;
global $ADODB\_COUNTRECS;
$ADODB\_COUNTRECS = false;
$start = microtime();
for ($i=0; $i < $max; $i++) {
$rs =& $db->Execute($sql);
$arr =& $rs->GetArray();
// print $arr[0][1];
}
$end = microtime();
$start = explode(' ',$start);
$end = explode(' ',$end);
//print\_r($start);
//print\_r($end);
// print\_r($arr);
$total = $end[0]+trim($end[1]) - $start[0]-trim($start[1]);
printf ("

seconds = %8.2f for %d iterations each with %d records

",$total,$max, sizeof($arr));
flush();
//$db->Close();
}
include("testdatabases.inc.php");
?>
